# Genotype distribution characteristics of multiple human papillomavirus in women from the Taihu River Basin, on the coast of eastern China

**DOI:** 10.1186/s12879-017-2328-6

**Published:** 2017-03-23

**Authors:** Jing-fen Lu, Guo-rong Shen, Qiong Li, Xu Chen, Chun-fang Ma, Tong-hua Zhu

**Affiliations:** Department of Clinical laboratory, Wujiang First People’s Hospital, Suzhou, People’s Republic of China

**Keywords:** Human papillomavirus, Cervical cancer, Genotypes, Distribution

## Abstract

**Background:**

There is limited data on the genotype distribution of human papillomavirus (HPV) in the Taihu River Basin, home to 1.29 million people on the coast of eastern China. This study evaluated the prevalence and genotypes among different female age groups in this region.

**Methods:**

Twenty-six HPV strains (low-risk HPV 6, 11, 40, 42, 44, 61, 73 and high-risk HPV 16, 18, 26, 31, 33, 35, 39, 45, 51, 52, 53, 55, 56, 58, 59, 66, 68, 82, and 83) were detected using Tellgenplex™ HPV technology in samples obtained from three clinical hospitals located in different regions of the Taihu Lake Basin.

**Results:**

The results showed that 1855 samples (20.97% of all samples) were found to be HPV-positive. Of these, 1375 samples (15.55% of all samples) were found to have a single HPV infection. Age-specific prevalence showed two peaks, one that corresponded to the group of 21–30 year-old women and the other peak that corresponded to the group of women over 51 years old. The three most prevalent genotypes were HPV52 (19.95%, 370/1855), HPV16 (13.48%, 150/1855), and HPV58 (11.32%, 210/1855). Mixed strains HPV58 + HPV33 and HPV58 + HPV52 were most commonly found in females infected with multiple HPV types.

**Conclusions:**

This investigation reveals that HPV infection in the Taihu River Basin varied significantly among different age groups. The most prevalent genotypes are included in the nonavalent vaccine, V503, however this vaccine is not licensed for use in mainland China. The most frequently occurring genotypes should be considered in the development of next-generation HPV vaccines for optimal protection of public health.

**Electronic supplementary material:**

The online version of this article (doi:10.1186/s12879-017-2328-6) contains supplementary material, which is available to authorized users.

## Background

Cervical cancer (CC) is a major cause of morbidity and mortality among women in China, and also represents an important global public health problem. Persistent infection by high-risk human papillomavirus (hrHPV) is required for the development of pre-invasive and invasive carcinogenesis [1, 2]. To date, more than 200 different viral types have been identified, and 40 of these types can infect the mucosa of the anogenital tract [3]. To some extent, improved detection of hrHPV may reduce the risk for the development of CC and associated deaths [4].

Early studies demonstrated that the distribution of HPV genotypes in CC varies with geographic location [2, 5, 6]. Hence, an accurate assessment of the regional genotype distribution is very important for effective strategies to prevent CC. Several studies have assessed the prevalence of HPV strains in mainland China, such as studies focused on Yunnan and Shanghai [7, 8] and a study that examined the HPV distribution in 37 cities in China [9]. However, there is limited data on the genotype distribution in the Taihu River Basin, which is a largely agricultural and industrial coastal area in eastern China. HPV genotyping detection is used to study HPV strain-specificity to facilitate the implementation, monitoring, and evaluation of vaccination programs. The American Society for Colposcopy and Cervical Pathology (ASCCP) guideline indicated that cervical screening should start at the age of 21, and screening using both the HPV test and the Pap test should be performed every 5 years for women 30–65 years in age [10]. The aim of this study was to determine the distribution of HPV genotypes in the Taihu River Basin to facilitate a better understanding of the HPV virus and to predict the efficacy of a HPV vaccine.

## Methods

### Study population

The purpose of this investigation was to obtain data on the prevalence of HPV genotypes, including age-specific prevalence differences and the distribution of multiple HPV genotypes. This investigation analyzed samples obtained from 3 clinical hospitals located in different regions of the Taihu River Basin. From November 2013 and June 2015, a total of 9012 women aged 21–70 years old (14,280 subjects were invited, but 5268 patients did not participate in the screening for various reasons) attended a gynecological outpatient clinic and expressed interest in cervical cancer screening. Participants met the following selection criteria: a) had not had sexual intercourse, had not used vaginal medications, and had not washed in the previous 48 h; b) not currently pregnant; c) with no history of total uterus or cervix resection; d) a permanent resident of the region; and e) agreed to undergo the HPV test and participate in this study.

### Specimen collection

The cervico-vaginal cells at the transformation zone of the uterine cervix were collected from all participants by a gynecologist using a standard cytobrush according to the standard sampling procedure at the recruitment sites. Cells were resuspended in 3 ml sample transport medium (STM) for the Tellgenplex™ HPV DNA Test (Tellgen Life Science, Shanghai, China) and stored at 4 °C. According to the kit instructions, samples can be stored in STM for 1 month at 2–8 °C and 6 months at −20 °C. We stored all samples at 4 °C for 1 week prior to testing to obtain the most accurate results.

### Ethics statement

This investigation was approved by the Ethical Committee of Wujiang First Hospital affiliated with Nantong University (permit number: 201405). All the specimens and their corresponding information were obtained with written informed consent from the participants involved in the project.

### HPV Genotyping

The Tellgenplex™ HPV DNA Test uses one multiplex PCR test combined with Luminex technology to identify the 26 most common HPV types (−6, −11, −16, −18, −26, −31, −33, −35, −39, −40, −42, −44, −45, −51, −52, −53, −55, −56, −58, −59, −61, −66, −68, −73, −82, and −83). The procedure includes DNA extraction, PCR amplification, and bead-coated hybridization and was performed according to the manufacturer’s guidelines [9, 11]. Each assay required 10–20 pg/ml HPV DNA as template. Females who were negative for β-globin were excluded from analyses.

### Statistical analysis

All statistical analyses were performed using SPSS version 19.0 (IBM, Armonk, NY, USA). All genotypes from single and multiple infections were computed individually. A multiple-type infection was defined as one that was positive for at least 2 types. These data were also stratified by age (21–30 years, 31–40 years, 41–50 years, 51–60 years, and 61–65 years).

## Results

### Distribution characteristics of HPV genotypes

**Fig. 1:**
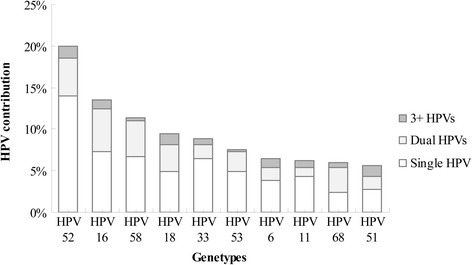
The top 10 HPV positive genotypes and their contribution

Among the 9012 individuals included in this study, 29 specimens were found to be negative for β-globin, and 138 cases could not be grouped because the analysis was restricted to women 21–65 old. Therefore, 8845 subjects (the median age was 42 and the quartile range was 32–47 years) were included in the analysis. As shown in Fig. 1, the overall prevalence of HPV was 20.97% (1855/8845), and 15.55% (1375/8845) of positive samples were found to have a single HPV infection, more than the number of subjects with a multiple infection. Among the study participants, 1430 had hrHPV infections, accounting for approximately 77% of the positive samples. The top 4 high-risk genotypes, 52, 16, 58, and 18, were detected in 19.95%, 13.48%, 11.32%, and 9.43% of positive specimens, respectively. HPV52 was the most prevalent type in subjects with a single HPV infection (260/1855 = 14.02%). Among the 480 individuals infected with multiple HPV types, 380 had dual infections (380/480 = 79.17%), and 100 subjects were infected by at least three HPV types (100/480 = 20.83%). The four most prevalent high-risk HPV types for subjects infected with multiple strains were HPV16, 52, 58, and 18, with frequencies of 6.20%, 5.93%, 4.58%, and 4.58% of positive samples, respectively.

### Prevalence of HPV infection in different age groups

**Fig. 2:**
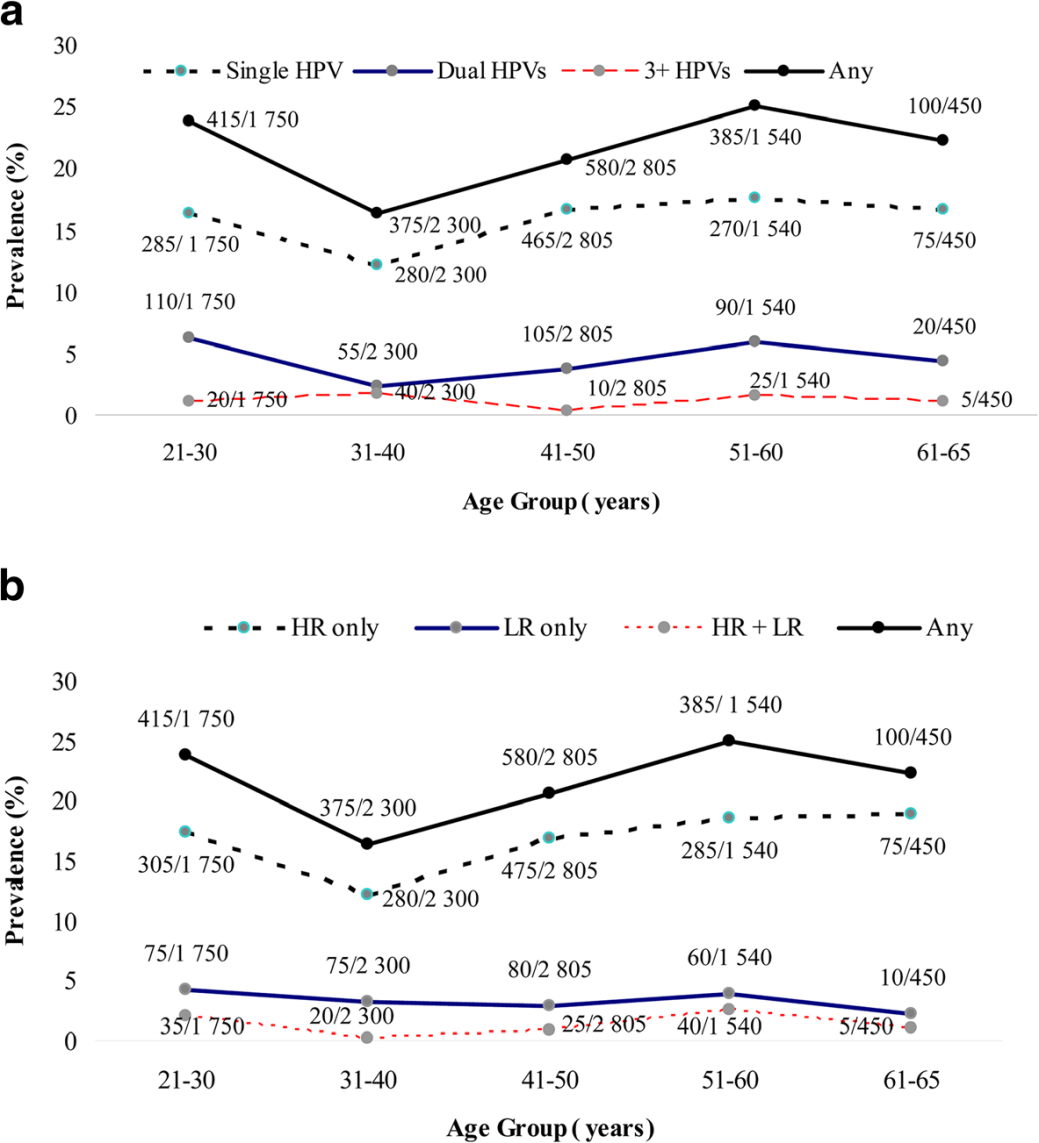
**a** Relative contribution of single and multiple HPV infections by age at diagnosis. **b** Relative contribution of high- and low-risk HPV infection by age at diagnosis

Among the age groups, the HPV infection prevalence ranged from 16.3% for women aged 31–40 years to 25% for those 51–60 yeas old (Fig. 2a, b). The frequency of hrHPV types also differed among these age groups. Similar to the overall prevalence of HPV, the highest overall prevalence of hrHPV was found in women 51–60 years old (16.67%, 75/450), and the lowest hrHPV prevalence was found in women 31–40 years old (12.17%, 280/2300; Fig. 2b). The prevalence for single and dual HPV infections exhibited two peaks, one for the group 21–30 years old and the other for the group 51–60 years old (Fig. 2a).

## Discussion

HPV infection is the most common sexually transmitted infection. The majority of HPV infections are temporary, especially among young women, but CC usually develops after 10 years of persistent hrHPV infection [12]. Regular gynecological screening and treatment of precancerous lesions are less effective strategies for the prevention of adenocarcinoma [13]. Fortunately, adenocarcinoma should be controllable by HPV vaccines [14]. Two different prophylactic HPV vaccines have been licensed and are now available in most countries except Mainland China. Obtaining a current dataset for a particular region will enable the development of optimal and effective screening and vaccination programs.

In this study, a rate of 20.97% HPV infection was observed in patients ranging from 21 to 65 years old. Most patients had a single infection. Of these HPV-positive patients, 77.09% were hrHPV infections, and there were two peaks in this analysis corresponding to women 21–30 years old and 51–60 years old. HPV is a sexually transmitted disease, and the higher rate of infection in younger, single women may be because these women may have more frequent sexual intercourse, particularly with more than one partner. Older women also show a higher rate of infection due to effects of physiological and immunological disorders associated with hormone fluctuations during the menopausal transition. These two peaks were also observed in the frequency analysis of multiple infections. Additionally, the frequency of dual and 3+ HPV infections was highest in individuals over 50 years old. These trends are consistent with the results of other researches that reported a second peak for the prevalence of multiple infections in females who are ≥50 or 60 years of age [8, 9, 12].

Of the three most dominant HPV genotypes, HPV 52, HPV 16, and HPV 58, 70.3%, 54%, and 59.5%, respectively, were single-infections and the remaining cases of these genotypes were present together with other HPVs. Wang et al. [15] reported that infections with multiple genotypes had higher risk of the development and progression of CC, but this does not necessarily mean that the cancerous stage requires multiple infections. Because cervical precursor lesions need years to develop into CC, multiple types may facilitate growth during the precancerous Cervical Intraepithelial Neoplasia (CIN) stages, but the oncogenic capability of the latter stages may not be controlled by multiple genotypes. In this present study, the top most frequent combinations of genotypes were HPV58 + HPV33 (32 cases) and HPV58 + HPV52 (25 cases) (Additional file 1: Table S1.). Both HPV52 and HPV58 are more prevalent in Eastern Asia, and the linking of HPV58 to cervical cancer was 1.8 higher than for HPV52 [16]. Further comparative analysis showed that HPV16, 52, and 58 are the three top hrHPV types in the Chinese reports, but the relative prevalence rates varied widely and other genotype distributions also differed regionally in Mainland [7, 17–19]. Therefore, infection with HPV58 or 52 in this region should be given the same attention as HPV16 and 18 infections. Fortunately, the V503 nonavalent vaccine appears to be safe and effective in the prevention of persistent infection and precancerous lesions associated with hrHPV types −16, −18, −31, −33, −45, −52, and −58, as well as genital warts that are related to lrHPV 6 and 11 infection [20]. This would cover 70.18% (965/1375) of single infections and 51.46% (247/480) of multiple infections in this area. However, this vaccine is not licensed in mainland China.

This analysis of HPV genotype distribution in the Taihu River Basin in China has potential limitations. First, this study determined the distribution of the prevalence of HPV genotypes in women aged 21–65 years who attended a cervical screening, however, the absence of cytological data of cervical pre-cancer or cancer remains a major concern. Second, there was no sampling of tissue specimens in this study. Comparison of HPV detection rates between cervical scraping and biopsy methods may help to clarify the pathological mechanism of HPV infection and the evolution of CC. Overall, this study provides a description of the genotype-specific prevalence in women from the Taihu River Basin on the coast of eastern China.

## Conclusions

Lifestyle and health are dramatically affected by economic conditions, cultural habits, and changing population dynamics. Therefore, it is urgent to pay attention to changes in the prevalence of HPV infection, particularly at a regional level. In the Taihu River Basin, the coast of eastern China, we found that the three top genotypes were HPV 52 (19.95%), HPV 16 (13.48%), and HPV 58 (11.32%). Mixed strains HPV58 + HPV33 and HPV58 + HPV52 were most frequently found in women with multiple infections. Considering the specific genotypes present in this geographic location, a cost-effective vaccine, such as the nonavalent vaccine V503, may provide optimal prevention of cervical cancer in this area.

## Additional file

Additional file 1: Table S1. Characteristics of HPV co-infection. (DOCX 14 kb)

## Abbreviations

CC: Cervical cancer
CIN: Cervical intraepithelial neoplasia
HPV: Human papillomavirus
hr-: High-risk
lr-: Low-risk
