# Supplementary material for: Genotype distribution characteristics of multiple human papillomavirus in women from the Taihu River Basin, on the coast of eastern China
Source: BMC Infect Dis. 2017 Mar 23;17:226. doi: 10.1186/s12879-017-2328-6 (PMC5364703; doi:10.1186/s12879-017-2328-6)
Supplement: Additional file 1: Table S1. — Characteristics of HPV co-infection. (DOCX 14 kb) [file 12879_2017_2328_MOESM1_ESM.docx]

**Additional file 1**

**Table S1** Characteristics of HPV co-infection

| Genotype | HPV52  (N=370) | HPV16  (N=250) | HPV58  (N=210) |
| --- | --- | --- | --- |
| HPV16 | 19 |  | 20 |
| HPV18 | 20 | 15 | 10 |
| HPV31 | 10 | 5 | 6 |
| HPV33 | 5 | 5 | 25 |
| HPV35 | 3 | 0 | 5 |
| HPV39 | 7 | 7 | 5 |
| HPV45 | 3 | 2 | 5 |
| HPV51 | 15 | 12 | 10 |
| HPV52 |  | 19 | 32 |
| HPV56 | 9 | 5 | 6 |
| HPV58 | 32 | 20 |  |
| HPV59 | 5 | 7 | 6 |
| HPV66 | 10 | 15 | 6 |
| HPV68 | 15 | 10 | 5 |

HPV, Human papillomavirus
